# Bioprospection of *Hura crepitans* metabolites against oxidative stress and inflammation: An *in vitro* and *in silico* exploration

**DOI:** 10.7150/ijms.109116

**Published:** 2025-03-12

**Authors:** Yu-Cheng Kuo, Bashir Lawal, Halimat Yusuf Lukman, Lung-Ching Chen, Sheng-Liang Huang, Yi-Fong Chen, Adewale O. Fadaka, Femi Olawale, Ayo Olasupo, Olabode T Ajenifujah, Dalia Fouad, Marios Papadakis, Gaber El-Saber Batiha, Saheed Sabiu, Alexander T.H. Wu, Hsu-Shan Huang

**Affiliations:** 1 Department of Pharmacology, School of Medicine, College of Medicine, Taipei Medical University, Taipei, Taiwan.; 2 School of Post-baccalaureate Chinese Medicine, College of Chinese Medicine, China Medical University, Taichung, Taiwan.; 3 UPMC Hillman Cancer Center, University of Pittsburgh, Pittsburgh, Pennsylvania.; 4 Department of Biotechnology and Food Science, Faculty of Applied Sciences, Durban University of Technology, P. O. Box 1334, Durban 4000, South Africa.; 5 Division of Cardiology, Department of Internal Medicine, Shin Kong Wu Ho-Su Memorial Hospital, Taipei 11101, Taiwan.; 6 School of Medicine, Fu Jen Catholic University, New Taipei 24205, Taiwan.; 7 Graduate Institute of Aerospace and Undersea Medicine, National Defense Medical Centre, Taipei 11490, Taiwan.; 8 Graduate Institute of Cancer Biology and Drug Discovery, College of Medical Science and Technology, Taipei Medical University, Taipei 11031, Taiwan.; 9 Department of Biotechnology, University of The Western Cape, Belleville, South Africa.; 10 Nano Gene and Drug Delivery Group, University of Kwazulu Natal, South Africa.; 11 Wonderful Institute for Sustainable Engineering, Chemical and Petroleum Engineering. University of Kansas.; 12 Department of Mechanical Engineering, Carnegie Mellon University, Pittsburgh PA, USA 15213.; 13 Department of Zoology, College of Science, King Saud University, PO Box 22452, Riyadh 11495, Saudi Arabia.; 14 Department of Surgery II, University Hospital Witten-Herdecke, Heusnerstrasse 40, University of Witten-Herdecke, 42283, Wuppertal, Germany.; 15 Department of Pharmacology and Therapeutics, Faculty of Veterinary Medicine, Damanhour University, Damanhour 22511, AlBeheira, Egypt.; 16 The Ph.D. Program of Translational Medicine, College of Medical Science and Technology, Taipei Medical University, Taipei, 11031, Taiwan.; 17 Clinical Research Center, Taipei Medical University Hospital, Taipei Medical University, Taipei 11031, Taiwan.; 18 Graduate Institute of Medical Sciences, National Defense Medical Center, Taipei 11490, Taiwan.; 19 Taipei Heart Institute, Taipei Medical University, Taipei 11031, Taiwan.; 20 School of Pharmacy, National Defense Medical Center, Taipei 11490, Taiwan.; 21 Ph.D. Program in Biotechnology Research and Development, College of Pharmacy, Taipei Medical University, Taipei 11031, Taiwan.

**Keywords:** *Hura crepitans*, antioxidant, anti-inflammatory, *in vitro*, *in silico*, phenolic compounds

## Abstract

**Background**: Despite the recognized therapeutic potential of *Hura crepitans*, its mechanistic antioxidant and anti-inflammatory actions remain underexplored.

**Methods**: This study investigates the inhibitory effects, binding stability, and interactions of metabolites from *H. crepitans* on oxidative and inflammatory biomarkers/targets using *in vitro* analyses and molecular dynamics (MD) simulations.

**Results**: *In vitro* experiments revealed significant dose-dependent antioxidant and anti-inflammatory activities. The crude methanolic extract (CMEHC) showed notable half-maximal inhibitory concentration (IC_50_) values for antioxidant assays, such as diphenyl picrylhydrazine (45.51 µg/mL) and ferric-reducing power (10.86 µg/mL), with comparable performance to standard ascorbic acid. Anti-inflammatory activities, including protein denaturation, proteinase inhibition, and membrane stabilization, demonstrated IC_50_ values between 77.29-171.30 µg/mL. Liquid chromatography-mass spectrophotometry identified five primary compounds, predominantly phenolics, with rutin as the most abundant. Computational analyses confirmed these compounds' safety profiles, robust binding interactions, and stability against oxidative and inflammatory targets, with rutin forming the most stable interactions.

**Conclusion**: These findings highlight the potential of *H. crepitans* phenolics as alternative therapies for oxidative stress and inflammation, warranting further drug development studies.

## Introduction

Vascular and organ abnormalities stemming from several reaction pathways that lead to free radical production and inflammatory responses are central to the pathogenesis of metabolic dysfunctions, including diabetes, cancer, cardiovascular, liver, kidney, neurodegenerative, and pulmonary diseases contributing significantly to global morbidity and mortality 1. In diabetes, oxidative stress and inflammation impair insulin signaling and secretion 2, while in cardiovascular diseases, oxidative stress and chronic inflammation exacerbate vascular endothelial dysfunction and atherosclerosis 3. Reactive oxygen and nitrogen species, such as superoxide and nitric oxide, activate pathways like cyclooxygenase and transcription factors such as NF-κB, driving disease progression 4. Addressing these pathways with effective antioxidant and anti-inflammatory agents is critical for therapeutic intervention.

Phenolics, naturally occurring compounds found in plants such as vegetables, fruits, and cereals, are recognized for their potent antioxidant and anti-inflammatory properties, with potential benefits for managing diabetes, cancer, and cardiovascular diseases 5. Although synthetic antioxidants like ascorbic acid and endogenous agents such as superoxide dismutase play a role in mitigating oxidative damage, limitations in cost, availability, and adverse effects often hinder their use. Similarly, non-steroidal anti-inflammatory drugs (NSAIDs) like aspirin are effective but not without risks 4. Consequently, there is a growing interest in plant-derived bioactive compounds as safer and more accessible alternatives.

*Hura crepitans L.*, commonly known as the "sandbox tree" or "monkey dinner bell," is a versatile member of the Euphorbiaceae family, valued for its traditional and medicinal uses across tropical regions of the Americas 7,8. This tree has long been recognized for its therapeutic properties, including its use as a laxative and remedies for inflammation, microbial infections, liver damage, leprosy, and as an emetic 9,10. Modern studies have validated these traditional applications, revealing a rich profile of bioactive compounds such as flavonoids, phenolic acids, alkaloids, tannins, carotenoids, terpenes, fatty acids, and essential amino acids like methionine and lysine 15-19. Notably, compounds such as rutin, myricetin, ferulic acid, and daphnane diterpenes contribute significantly to its pharmacological potential 9,20.

Research has demonstrated various pharmacological effects of *H. crepitans*, including hepatoprotective, antihypertensive, antidiabetic, antioxidant, and anti-inflammatory activities 16,21-25. However, while these findings underscore its therapeutic promise, the mechanisms underlying its antioxidant and anti-inflammatory effects remain poorly understood, particularly through computational approaches. Such methods, in combination with *in vitro* studies, have proven essential in modern drug discovery, providing efficient ways to explore the pharmacodynamics of medicinal plants and translate traditional knowledge into therapeutic innovations 26-32. Consequently, this study investigated the *in vitro* antioxidant and anti-inflammatory of *H. crepitans* and computationally explored its mechanism of action, setting the stage for future drug development endeavors.

## Materials and Methods

### Collection and preparation of the extract

*Hura crepitans* plant was collected within Minna metropolis, Niger state, Nigeria, identified and authenticated at the herbarium unit of the Federal University of Technology (FUT), Minna, Nigeria where a voucher number was deposited. Crude methanolic extract of *H. crepitans* (CMEHC) was obtained by firstly rinsing dirt and dust off the plant with clean water and air-dried until a constant dry weight was obtained. The dried plant material was then ground into a fine powder and extracted with methanol (1:5) for 72 h with intermittent shaking for complete extraction. Subsequently, the mixture was filtered using Whatman filter paper (No. 1), and the filtrate concentrated in a rotary evaporator. The CMEHC obtained was then stored in a refrigerator (4°C) for further use.

### *In vitro* antioxidant analysis

The antioxidant potential of the extract was evaluated by analyzing its DPPH radical-scavenging, ferric-reducing antioxidant power (FRAP), and lipid peroxidation (LPO) inhibitory potentials. For the DPPH assay, varying concentrations of CMEHC (50-400 µg/mL) and reference standard (ascorbic acid) were mixed with a DPPH solution in methanol and allowed to react in darkness for 45 min. The reduction in absorbance indicating DPPH scavenging activity was measured at 517 nm 33. In the FRAP assay, varying concentrations of CMEHC were reacted with a mixture containing potassium hexacyanoferrate (III) and trichloroacetic acid in sodium phosphate buffer. Following centrifugation, ferric chloride was introduced to the supernatant for color development, and absorbance was recorded at 700 nm 34. The LPO analysis involved treating a mixture of egg homogenate and FeSO_4_ with CMEHC, followed by incubation and subsequent addition of acetic acid and an acid-reactive substance. After heating, butanol was added, the mixture was centrifuged, and absorbance was read at 532 nm 35. The percentage inhibition was calculated using the equation displayed, while the half-maximal inhibitory concentration (IC_50_) was determined using linear regression.

Percentage inhibition = 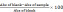


### *In vitro* anti-inflammatory analysis

The anti-inflammatory potential of the extract was investigated for its human red blood cell (RBC) membrane stability, protein denaturation inhibition, and proteinase inhibitory capabilities. For the RBC membrane stability assay, freshly collected human plasma was processed and mixed with reference standard aspirin or varying CMEHC concentrations in a 10% red blood suspension. Following incubation at 56 °C for 30 min and centrifugation, the absorbance of the supernatant was measured at 560 nm 36. The protein denaturation inhibition assay involved mixing CMEHC or aspirin with a 1% bovine serum albumin (BSA) solution. The mixture was heated at 55 °C for 30 min and allowed to cool to observe turbidity 37. For the proteinase inhibitory assay, a reaction mixture containing trypsin, Tris-HCl buffer, and CMEHC was incubated, followed by the addition of casein and further incubation. The reaction was terminated with perchloric acid, and after centrifugation, the absorbance of the supernatant was measured at 210 nm against a Tris-HCl buffer 38. The percentage inhibition was calculated using the equation displayed, while the half-maximal inhibitory concentration (IC_50_) was determined using linear regression.

Percent inhibition = 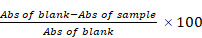


### Characterization of the CMEHC with a liquid chromatographic mass spectrometric (LC-MS) analysis

The LC-MS analysis of the crude methanolic extract of *Hura crepitans* (CMEHC) was done using a Shimadzu LC-MS-8040 ultrafast mass spectrometer, equipped with a Shim-pack FC-ODS analytical column. The analysis involved a dual mobile phase system: mobile phase A with 5 mmol/L ammonium acetate in water and mobile phase B with 5 mmol/L ammonium acetate in methanol. The gradient elution program varied the concentration of phase B from 15% to 95% over a 40-minute period, maintaining a flow rate of 0.2 mL/min and a column temperature of 40 °C. Mass spectrometry conditions included a +4.5 kV probe voltage in ESI-positive mode, nebulizing gas flow of 1.5 L/min, drying gas of 10 L/min, with temperatures set at 250 °C for the drying gas and 400 °C for the heat block. Scans ranged from 100 to 1000 m/z at a speed of 5000 u/s in the positive ionization mode, with spectra monitored using Shimadzu Lab Solution software over a 0.00-50.00 retention time frame. Data analysis was conducted in stages, starting with mass detection and chromatogram construction, followed by peak deconvolution and filtering using mzmine software (version 2.53) for compound identification, after exporting the results in CDF format for enhanced peak analysis efficiency 39,40.

### *In silico* pharmacokinetics studies

The pharmacokinetics (PK), drug-likeness, medical, and physicochemical properties of the most abundant biologically active compounds of CMEHC were assessed using the ADMERLab, ADMETSar, and SWISSADME online databases 41.

### Molecular docking and dynamics simulation

**Figure 1:**
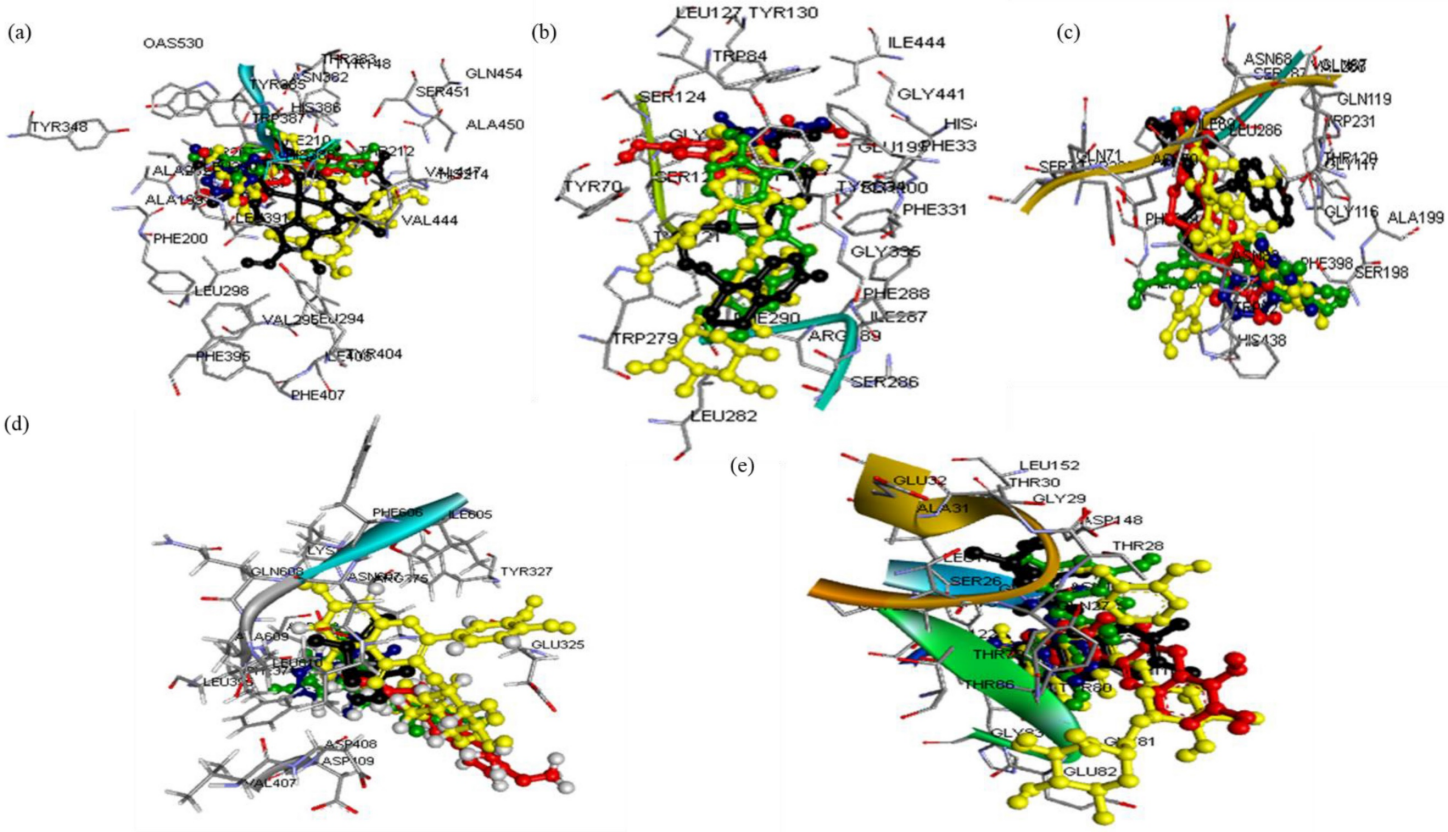
Super-imposed structure of the docked phenolic compounds [rutin (yellow), dihydroberberine (red), epigallocatechin (green)] of crude methanolic extract of *H. crepitans* and reference standard; aspirin/ascorbic acid (blue) within the catalytic amino acid residues of (a) COX-2 (b) AChE (c) BChE (d) NF-kB (e) NOx on their native inhibitor (black). All had the same RMSD value of < 1.0 Å.

The three-dimensional (3D) structures of the ligands (rutin, dihydroberberine, and epigallocatechin) were acquired using the Avogadro molecular builder and visualization tool (version 1.XX), initially in mol2 format, and then converted to PDB format with the PyMOL Molecular Graphics System (version 1.2r3pre). Inflammatory hub targets [COX-2 (5F19), acetylcholinesterase (1H23), butyrylcholinesterase (5LKR), nuclear factor-κB [NF-κB (1NFK), and NADPH oxidase (NOx] were sourced from the Protein Data Bank (PDB) in PDB format 42. These targets underwent preparation for docking and saved as PDBQT files for compatibility 43-45. The docking process utilized Avogadro software, and the results visualized in both PyMOL and Discovery Studio Visualizer (version 19.1.0.18287, BIOVIA, San Diego, CA, USA) 44,46-48. Docking validation was conducted to prevent pseudo-positive binding by ensuring superimposition of the docked phenolic compounds at the target's active site as the native ligands with root mean square deviation (RMSD) of 0.5 Å for each target with the visualization done using Discovery Studio v21.1.0 23 (**Figure 1**).

Molecular dynamics (MD) simulations of the docked rutin complexes were executed using the Schrodinger suite (2020-2), specifically the Desmond software integrated within the system builder module of Maestro (version 12.4). These simulations spanned a 100 ns timeframe, adhering to methodologies detailed in prior studies 49,50. Post-simulation analyses including root-mean-square fluctuation (RMSF), root-mean-square deviation (RMSD), radius of gyration (rGyr), solvent-accessible surface area (SASA), changes in secondary structure, and hydrogen bond counts 51 were analyzed. The binding free energy of the complexes was calculated employing the molecular mechanics Poisson-Boltzmann surface area (MM-PBSA) method, analyzing 1000 trajectory files to ensure thorough assessment.

### Data analysis

Data were analyzed using GraphPad Prism software. All values are expressed as the mean ± standard error of the mean (SEM) of three independent replicates (n = 3) to reflect the consistency and reliability of the experimental outcomes. The IC_50_, defined as the concentration required to achieve 50% inhibition of the targeted activity, was determined and compared between the extract and standard compounds.

## Results

### *In vitro* antioxidant and anti-inflammatory effects of the CMEHC

**Figure 2:**
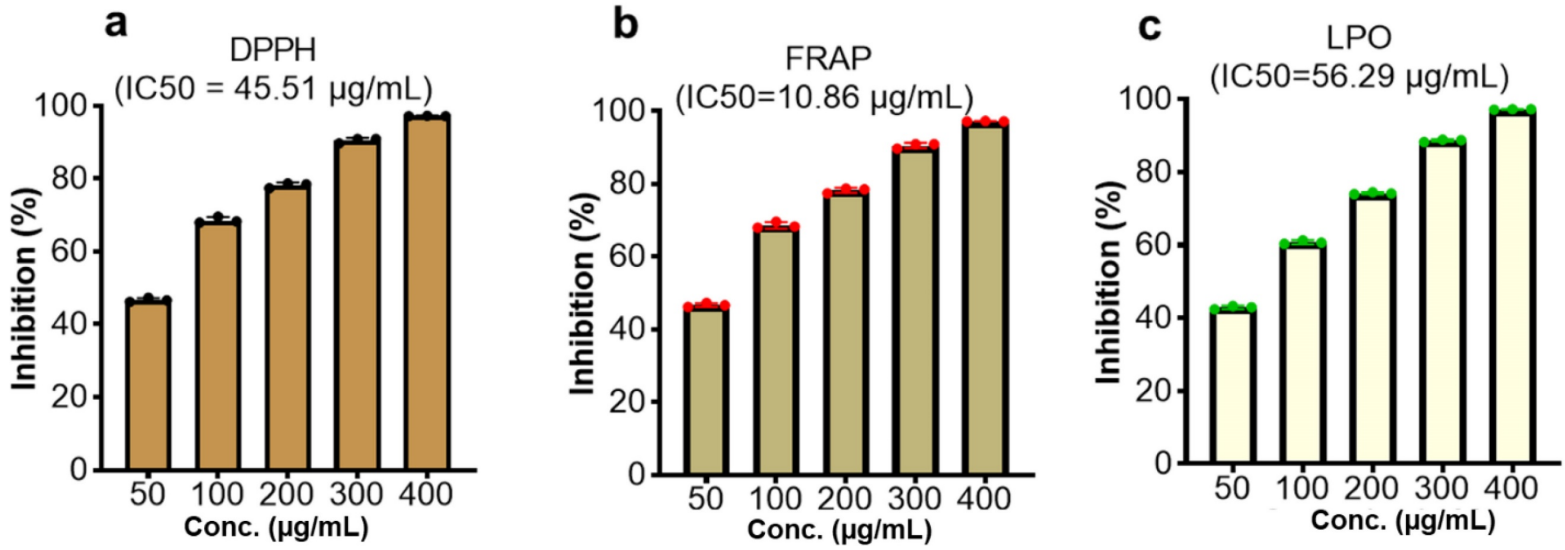
Effect of the CMEHC on (a) 2,2-diphenyl-1-picrylhydrazyl (DPPH) (b) ferric-reducing antioxidant power (FRAP), and (c) lipid peroxidation (LPO) IC_50_, 50% inhibitory concentration. Values are presented as the mean ± standard error of mean of the replicates (*n*=3). IC_50_, 50% inhibitory concentration.

The evaluation of the CMEHC for its antioxidant and anti-inflammatory potential demonstrated a dose-dependent activity across the various assays. For antioxidant activity, the extract showed significant inhibition in the DPPH, FRAP, and lipid peroxidation (LPO) assays, with IC_50_ values of 45.51 µg/mL, 10.86 µg/mL, and 56.29 µg/mL (**Figure 2**), respectively while IC_50_ values of 20.90 µg/mL, 15.32 µg/mL, and 21.87 µg/mL, respectively, were recorded for ascorbic acid. The highest inhibition percentages for ascorbic acid were recorded at a concentration of 250 µg/mL, demonstrating values of 98.46 ± 0.15%, 98.13 ± 0.02%, and 98.69 ± 0.13% for each assay, respectively.

**Figure 3:**
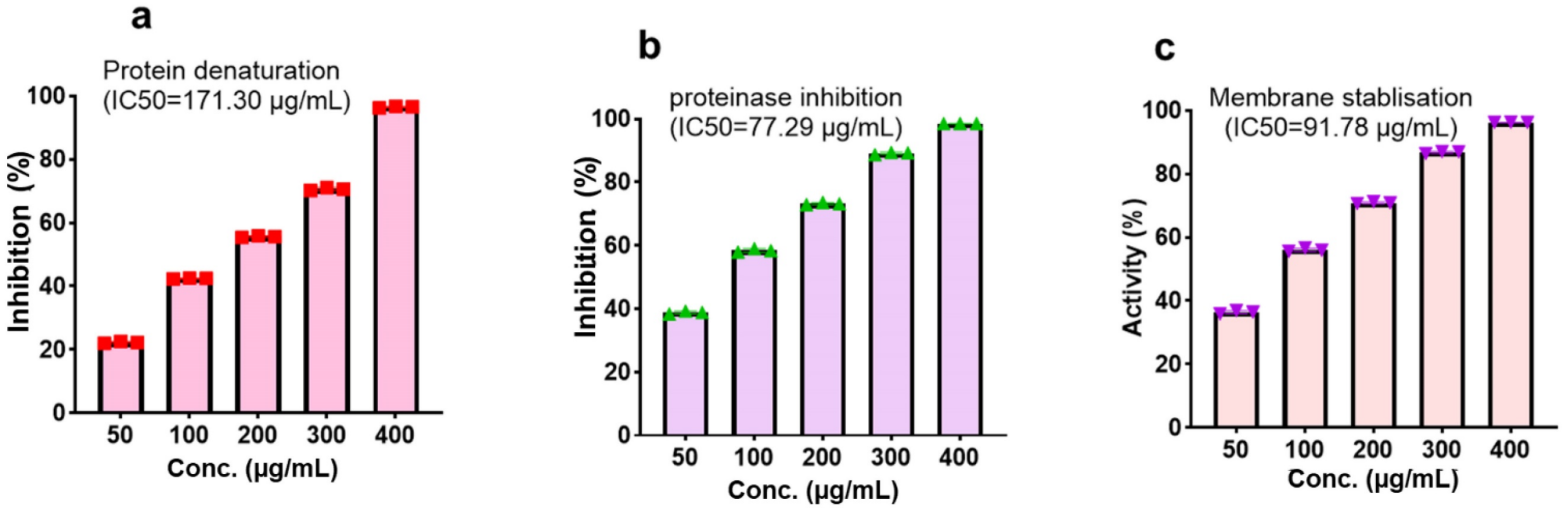
Effect of the CMEHC on (a) protein denaturation, (b) proteinase inhibition, and (c) membrane stabilization. IC_50_, 50% inhibitory concentration. Values are presented as the mean ± standard error of mean of the replicates (*n*=3). IC_50_, 50% inhibitory concentration.

With regards to the anti-inflammatory activity, CMEHC exhibited dose-responsive effectiveness in inhibiting protein denaturation, proteinase inhibition, and stabilizing cell membranes, with IC_50_ values of 171.30 µg/mL, 77.29 µg/mL, and 91.78 µg/mL (**Figure 3**)., respectively. Aspirin had inhibition percentages of 96.86 ± 0.08%, 99.80 ± 0.01%, and 97.76 ± 0.15% at a concentration of 250 µg/mL, and presented IC_50_ values of 39.40 µg/mL, 8.15 µg/mL, and 10.12 µg/mL for the respective assays.

### High-Performance LC-MS Analysis of the Bioactive Compounds in CMEHC

**Figure 4:**
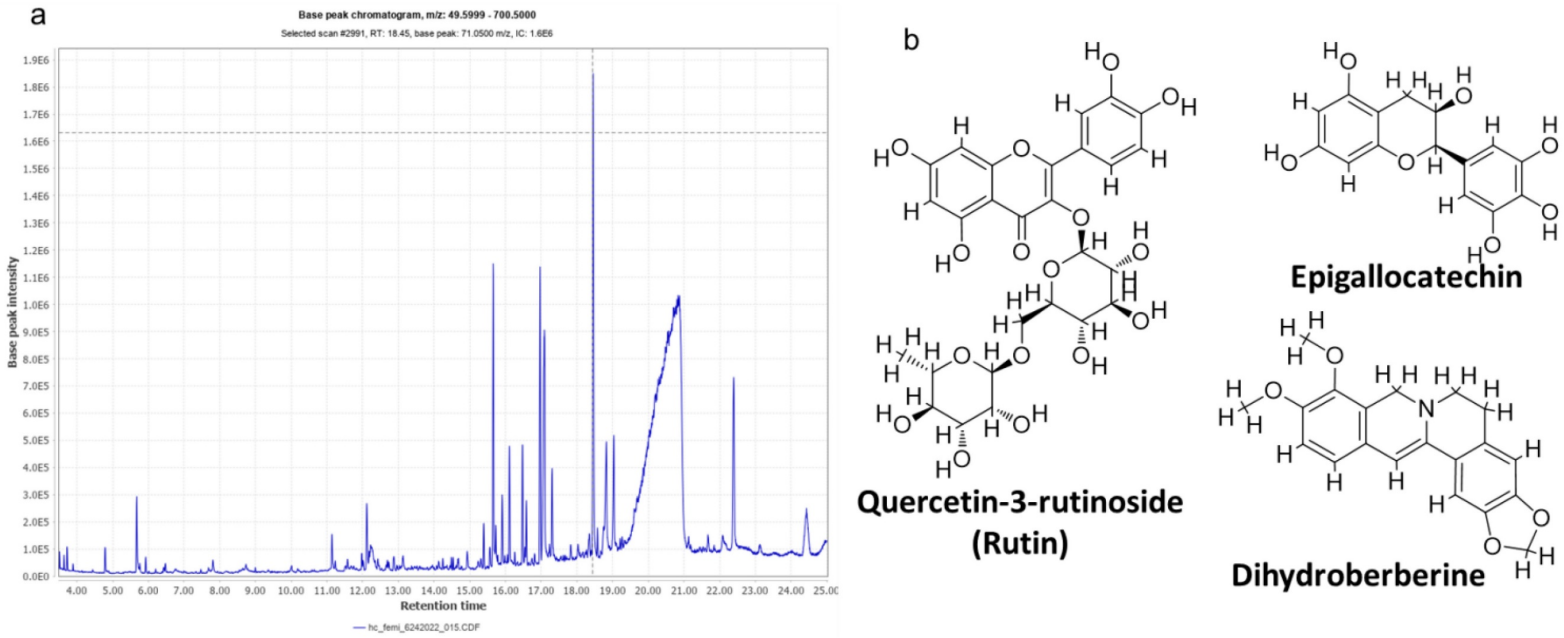
** (a)** LC-MS chromatogram of the crude methanolic extract of *Hura crepitans* (CMEHC), illustrating the retention times and intensities of the identified bioactive compounds. **(b)** Two-dimensional structures of epigallocatechin, quercetin-3-rutinoside, dihydroberberine, 2-(3,4-dihydroxyphenyl)-5,7-dihydroxychromene-4-one identified.

The high-performance LC-MS analysis of CMEHC identified five principal compounds, with their mass detection facilitated by mzmine software, applying a retention time (RT) tolerance of 0.01 min and a mass-to-charge ratio (m/z) tolerance of 0.02 or 5.0 ppm. These compounds were identified using comprehensive libraries and databases, presenting a sequence of increasing percentage areas: epigallocatechin, quercetin-3-rutinoside, dihydroberberine, 2-(3,4-dihydroxyphenyl)-5,7-dihydroxychromene-4-one, and hexadecanoic acid. The RT values observed were 25.234 min for epigallocatechin, 14.139 min for quercetin-3-rutinoside, 21.480 min for dihydroberberine, 26.433 min for 2-(3,4-dihydroxyphenyl)-5,7-dihydroxychromene-4-one, and 24.926 min for hexadecanoic acid. Quercetin-3-rutinoside exhibited the highest base peak value (m/z) of 61.11 ppm, followed by dihydroberberine (33.825 ppm), epigallocatechin (30.425 ppm), 2-(3,4-dihydroxyphenyl)-5,7-dihydroxychromene-4-one (28.435 ppm), and hexadecanoic acid (256.30 ppm) (**Table 1**). The chromatogram and structural elucidation of the phenolic compounds is represented (**Figure 4a, b**).

### *In silico* analysis of the pharmacokinetics and drug-likeness properties of selected CMEHC compounds

The *in-silico* evaluation of three phenolic compounds from CMEHC—rutin, dihydroberberine, and epigallocatechin—focused on their pharmacokinetic (PK) profiles, drug-likeness, and physicochemical characteristics (**Table 2**). The analysis revealed that all compounds exhibited satisfactory bioavailability scores and intestinal absorption, except for rutin. Besides rutin, both dihydroberberine and epigallocatechin had molecular weights less than 500 g/mol and demonstrated synthetic accessibility scores below 400. The trio showed high plasma protein binding rates (over 80%) and extensive volume of distribution. Notably, dihydroberberine alone was identified as blood-brain barrier permeable. Despite these differences, all compounds were characterized by favorable half-lives and clearance rates, alongside minimal interaction with cytochrome P450 isoenzymes, either as inhibitors or substrates.

#### Molecular docking analysis reveals rutin as a potent inhibitor of neuronal and anti-inflammatory targets

**Figure 5:**
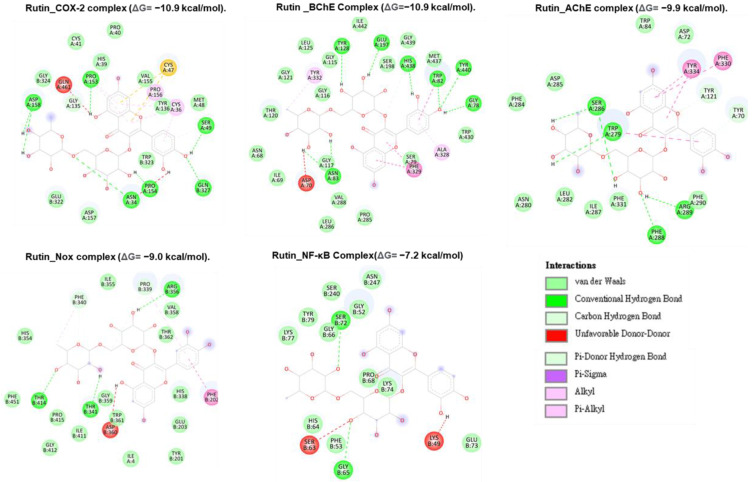
Two-dimensional representation of rutin's molecular interactions with key targets involved in oxidative stress and inflammation: cyclooxygenase-2 (COX-2), acetylcholinesterase (AChE), butyrylcholinesterase (BChE), nuclear factor-kappa B (NF-κB), and NADPH oxidase (Nox). The interactions highlight rutin's binding sites, hydrogen bonds, hydrophobic interactions, and other key molecular interactions, demonstrating its potential to modulate these targets' activity and contribute to its antioxidant and anti-inflammatory effects.

**Figure 6:**
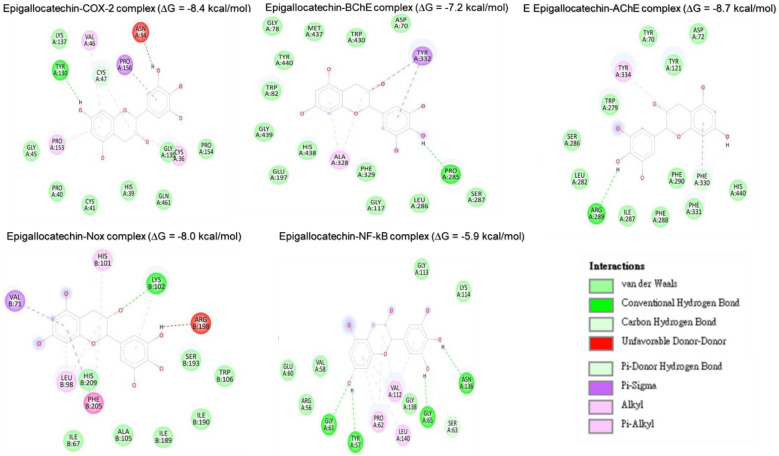
Two-dimensional representation of epigallocatechin's molecular interactions with critical targets involved in oxidative stress and inflammation: cyclooxygenase-2 (COX-2), acetylcholinesterase (AChE), butyrylcholinesterase (BChE), nuclear factor-kappa B (NF-κB), and NADPH oxidase (Nox). The depiction illustrates binding sites, hydrogen bonds, hydrophobic interactions, and other key molecular interactions, underscoring epigallocatechin's potential to modulate these targets and contribute to its therapeutic antioxidant and anti-inflammatory properties.

**Figure 7:**
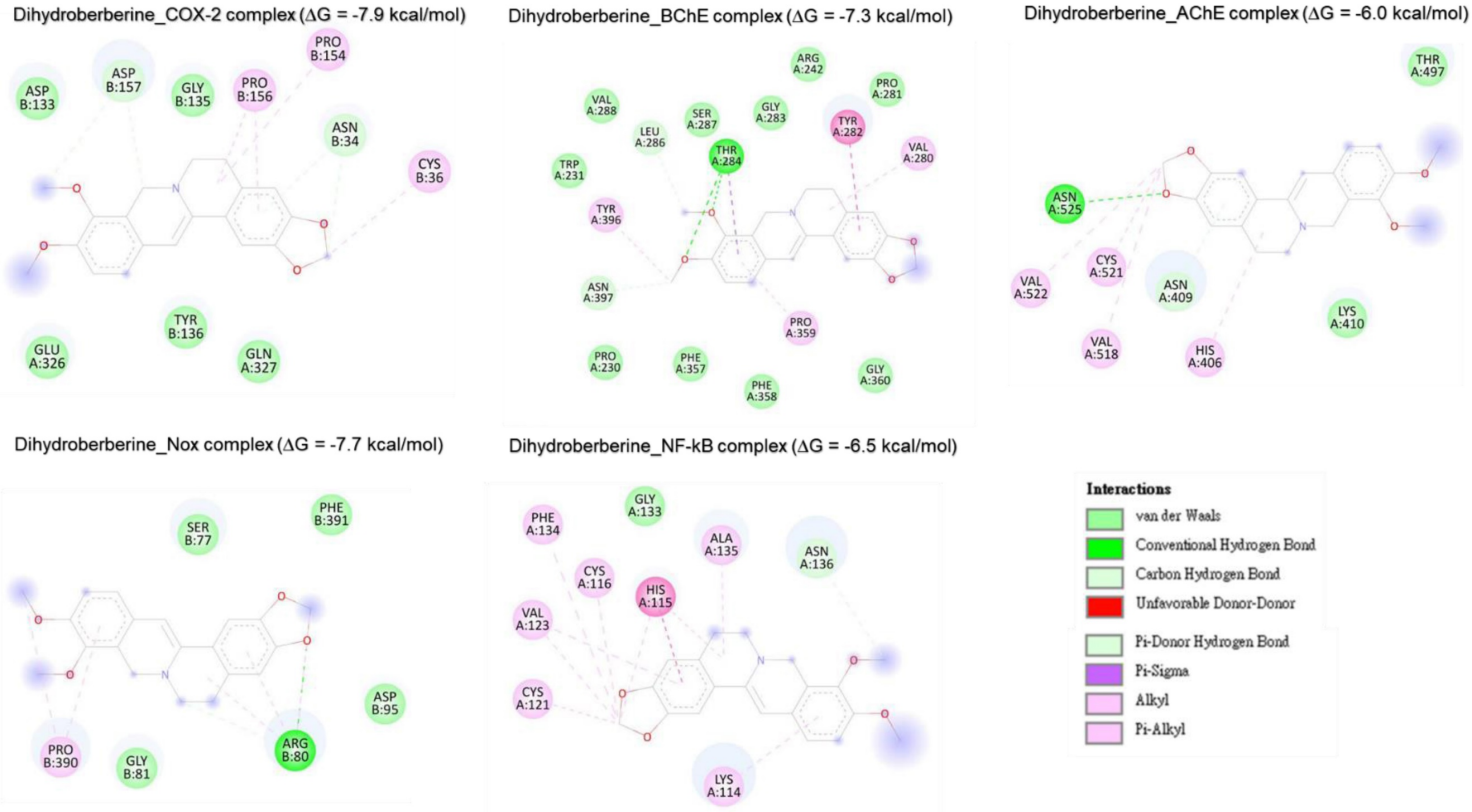
Two-dimensional representation of dihydroberberine 's molecular interactions with critical targets involved in oxidative stress and inflammation: cyclooxygenase-2 (COX-2), acetylcholinesterase (AChE), butyrylcholinesterase (BChE), nuclear factor-kappa B (NF-κB), and NADPH oxidase (Nox). The depiction illustrates binding sites, hydrogen bonds, hydrophobic interactions, and other key molecular interactions, underscoring epigallocatechin's potential to modulate these targets and contribute to its therapeutic antioxidant and anti-inflammatory properties.

**Figure 8:**
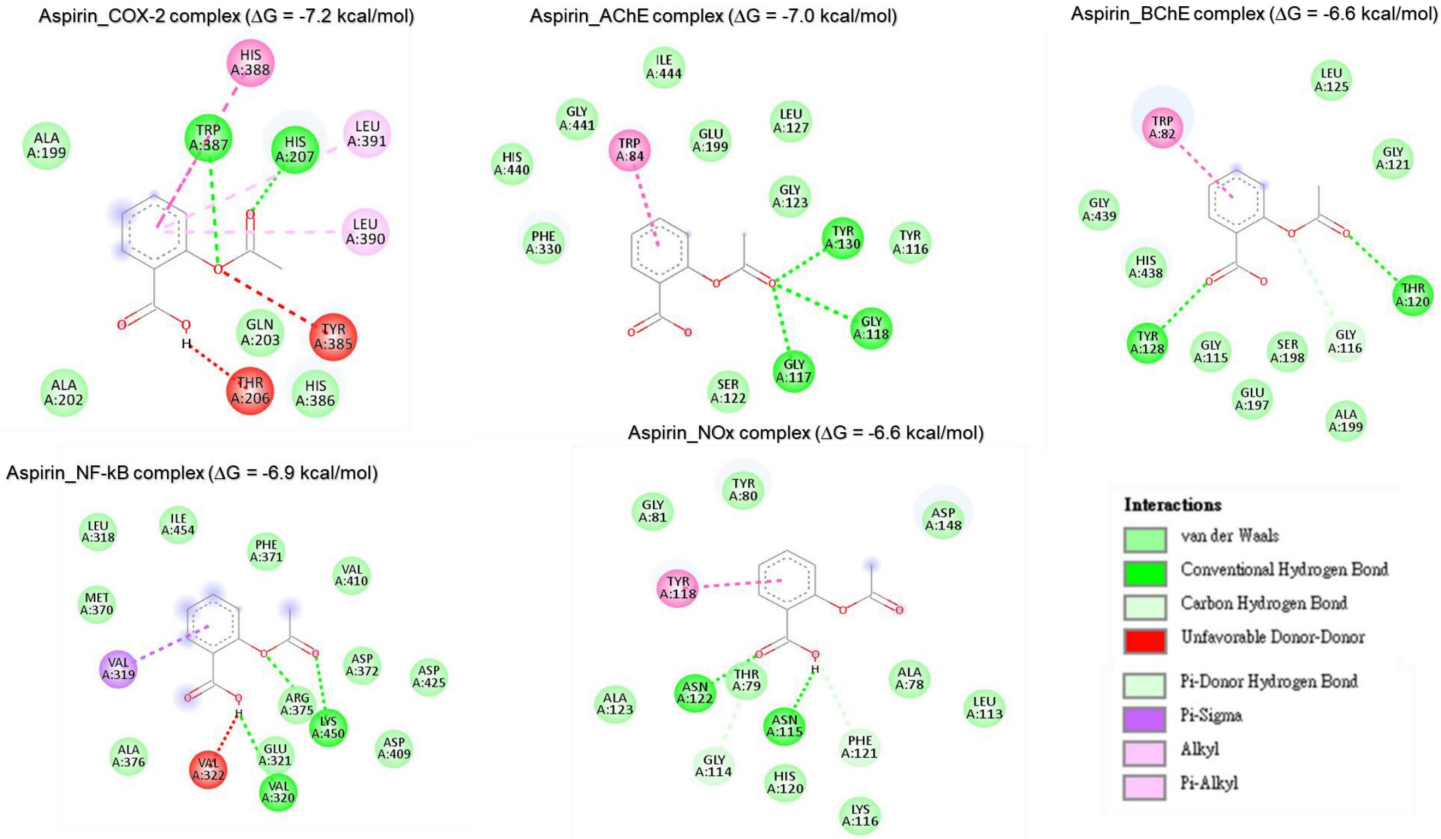
Two-dimensional representation of aspirin's molecular interactions with critical targets involved in oxidative stress and inflammation: cyclooxygenase-2 (COX-2), acetylcholinesterase (AChE), butyrylcholinesterase (BChE), nuclear factor-kappa B (NF-κB), and NADPH oxidase (Nox).

Molecular docking studies were performed to evaluate the interaction between CMEHC compounds (rutin, dihydroberberine, and epigallocatechin) and key neuronal and inflammatory targets, including [cyclooxygenase (COX-2), acetylcholinesterase (AChE), butyrylcholinesterase (BChE), nuclear factor-kappa B (NF-κB), and NADPH oxidase] (**Figures 5-7**). The reference standard (aspirin) binding with the targets are shown in **Figure 8**. Except for AChE-dihydroberberine and NF-kB-epigallocatechin complexes with lower negative docking score that the standards, phenolic compounds in CMEHC presented higher negative docking scores than the standards when docked with the investigated targets (**Table 3**). Comparatively, the compounds exhibited strong binding affinities to the target proteins, with COX-2 emerging as the most vulnerable and NF-κB as the least (**Table 3**). Among the compounds, rutin displayed the most significant binding efficiency, with the lowest docking scores (ΔG) ranging from -7.2 to -10.9 kcal/mol, relative to the epigallocatechin (ΔG= -5.9 to -8.7 kcal/mol) and dihydroberberine (ΔG= -6.0 to -7.9 kcal/mol). Binding interactions between the targets and the compounds showed several interacting amino acids including H-bonding, alkyl interactions pi-interactions and several van der Waal forces with rutin again having more interacting amino acid residues with the targets (**Table 4**). The superior binding affinity of rutin was further confirmed by its higher MMGBSA scores (**Table 5**), ranging from -38.16 to -61.65 kcal/mol, compared to epigallocatechin (-20.30 to -34.60 kcal/mol) and dihydroberberine (-31.29 to -47.36 kcal/mol). These findings, corroborated by various docking models, pinpoint rutin as the leading candidate for targeting these targets. Consequently, rutin was chosen for further exploration through molecular dynamics (MD) simulation.

### Molecular dynamics simulation of top ranked *H. crepitans* phenolics with anti-inflammatory proteins

**Figure 9:**
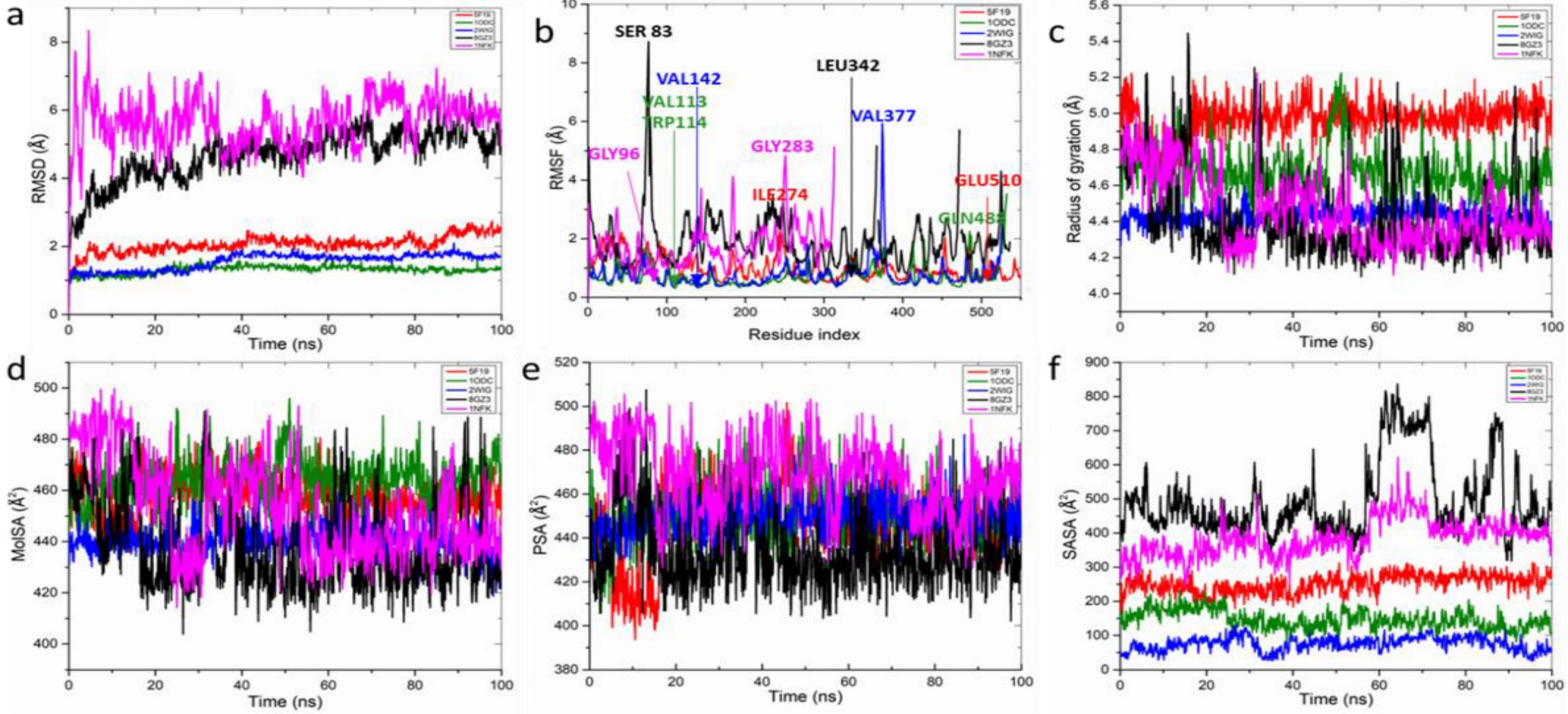
Post dynamics plots of (a) root mean square deviation (RMSD) plot of active compound-protein complexes in 100 ns, which is made up of α-carbon (Cα) atoms, throughout the simulations. (b) root mean square fluctuation (RMSF) plot of active compound-protein complexes during 100-ns MDs. Compound-protein target interaction properties including (c) radius of gyration (rGyr) (d) the molecular surface area (MolSA), (e) polar surface area (PSA), and (f) solvent-accessible surface area (SASA) of the hit compound of CMEHC with key anti-inflammatory targets. Each color represents different protein complexes with rutin. Red-cyclooxygenase-2 + rutin; olive, acetylcholinesterase + rutin; blue, butyrylcholinesterase + rutin; black, NADPH oxidase + rutin; and pink, nuclear factor-κB + rutin

The structural dynamics and interactions of rutin which demonstrated the lowest free binding energy when complexed with key anti-inflammatory targets were analyzed (**Figure 9** and **Table 6)**. The RMSD plot showed stable rutin-target patterns with AChE-rutin complex having fluctuated higher relative to the other complexes (**Figure 9a**) over the 100-ns simulation period, displaying a range of RMSD values: AChE (1.31±0.12) < BChE (1.55±0.24) < COX-2 (2.06±0.25) < NOx (4.68±0.76) < NF-kB (5.67±0.70). The RMSF plot varying fluctuation peak patterns in each target complex (**Figure 9b**), with mean values spanning 0.71±0.38 to 1.89±0.87 Å (**Table 6**). Notably, certain residues exhibited significant interactions with rutin, demonstrating minimal fluctuations, such as GLU510 in COX-2 and VAL113 and TRP114 in AChE, whereas others, like ILE274 in COX-2 and GLN488 in AChE, showed greater fluctuations due to lesser interactions (**Table 6**).

The rGyr plot demonstrated initial instability across all systems within the first 20 ns, however, formed stable complexes from 60 ns to the end of the simulation **(Figure 9c)** with slightly different mean values ranging from 4.41±0.22 to 4.96±0.09 Å^2^ for all the targets (**Table 6**). With regards, the molecular surface area (MolSA) and polar surface area (PSA), minimal fluctuating plots were observed in all the target-rutin bound complexes (**Figure 9d, e**) with NOx-rutin complex having the least mean values of 438.26±18.16, 433.79±17.39 Å^2^ among all the targets (**Table 6**). However, increasing fluctuating SASA was observed (**Figure 9e**) exhibiting significantly different mean values with NOx having the highest mean SASA value of 496.11±109.47 Å^2^ (**Table 6**).

## Discussion

Oxidative stress and inflammation result in dysfunctions in lipid, nucleic acid, protein, and carbohydrate metabolism 52 with effects culminating in cellular/organ failure and subsequent morbidity and mortality risk 53. This is because the incessant assault of biological molecules by free radicals produced during oxidative stress and inflammatory responses are precursors to numerous diseases. The defense against these radicals is fortified by antioxidants, which mitigate their harmful effects 54. The myriads of therapeutic benefits of medicinal plants as alternative drug agents have been recognized and documented extensively, highlighting their pivotal role in combating various ailments through ages 55. This study elucidated the antioxidant and anti-inflammatory capabilities of *Hura crepitans*, employing a combination of *in vitro* and *in silico* methodologies.

Medicinal plants have historically been a cornerstone in the treatment of diverse health conditions, owing to their rich repository of phytochemicals that underpin their therapeutic efficacy 56. The *in vitro* antioxidant analysis of CMEHC showcased its potent DPPH-inhibitory effect, FRAP, and LPO, protein denaturation, proteinase and enhanced membrane stability. The observed lower IC_50_ values in the standards relative to the extract might be due to their pure and refined state which might influence their affordability and availability in contrast to herbal medications. The protective capabilities of plants against these pathways, as evidenced by their ability to counteract inflammation and inhibit protease activities, are crucial 13. Our findings align with these observations, as CMEHC inhibited protein denaturation, proteinase activity, and membrane destabilization effectively, supporting its potential as a dual-functional therapeutic agent, mirroring the *in vivo* anti-inflammatory and antidiabetic study of Lawal et al. 30.

Complementing these observations, Vassallo et al. 13 identified key compounds such as epigallocatechin in *H. crepitans*, with epigallocatechin showing promise in mitigating oxidative and endoplasmic reticular stress, among other biological activities 57. This aligns with our findings where CMEHC compounds not only demonstrated significant pharmacokinetic and drug-likeness properties but also indicated their suitability for oral therapeutic applications.

Consequent of the findings from the *in vitro* study, the potential anti-inflammatory mechanism of *Hura crepitans* metabolites were investigated for possible hit compounds. Additionally, delving into the molecular mechanisms through protein-ligand interactions, a critical component in myriad biological processes, offered further insights on the biological mechanism of action of metabolites 58. While virtual screening informs the pharmacokinetics, drug-likeness, synthetic ability, and toxicity profiles of a compound, molecular docking and MD simulation provides information on the binding fitness, and binding stability and interactions, respectively 59,60.

Naturally occurring antioxidants contribute significantly to the health-promoting properties of plants, capable of donating hydrogen atoms to neutralize the destabilizing effects of free radicals in reactions such as DPPH and LPO, besides catalyzing reduction processes 61,62. Due to the relationship between oxidative stress and inflammation 52, there is a high chance of a molecule to affects both conditions. On the premise of phenolics being potent antioxidant and anti-inflammatory agents 63, the identified phenolic compounds in the extract were subjected to *in silico* analysis. The three phenolic compounds demonstrated significant binding fitness and orientation with the targets better than the reference standard and further underscores the capability of *H. crepitans* modulatory effect on the investigated targets and as safer and more cost-effective alternative antioxidant and anti-inflammatory agents.

Although rutin demonstrated lesser pharmacokinetics, drug-likeness properties relative to the other two compounds, it presented greater binding fitness. Based on this, rutin was subsequently selected for MD simulations. Furthermore, reactions leading from free radical generation to inflammatory responses, mediated through mechanisms like RBC peroxidation and lysosomal membrane destabilization, underline the importance of anti-inflammatory agents 64-66. Thus, rutin binding interaction was analyzed with neuronal inflammatory targets including COX-2, AChE, BChE, NADPH oxidase, and NF-κB to solidify its anti-inflammatory potential, dictated by low binding energies and favorable non-covalent interactions observed in molecular docking studies. The free binding energy describes the binding affinity of a compound to the active site of a target with lower free binding energy indicating better binding affinity and pointer to the level of stability of the complex 67. The simulations hinted the stability and minimal conformational changes of rutin, especially notable in complexes with COX-2, AChE, and BChE, throughout the 100-ns simulation period.

Post dynamics parameters such as RMSD, RMSF, and rGyr describe stability of a complex in term of convergence, interaction of the compound in the binding pocket of the target, and complex compactness, respectively 68. With regards RMSD, the stability is inversely proportional to the binding stability 69. The RMSD analysis indicated that the complexes remained stable over the 100-ns simulation period with Nox- and NF-kB-rutin complexes having > 3.0 limit reported for a stable complex 70 indicative of lesser stability with rutin. The observed stability of the rutin-target bond complexes is consistent with the free binding energy for all the complexes where NOx with lower free binding energy had high mean RMSD and hence lesser stability. The flexibility of a protein's binding pocket upon binding of a compound suggests the stability of the complex formed relative to the important amino acids in the active site of the protein and 71. Notably, certain residues in the active site of the investigated targets exhibited reduced fluctuations suggestive of significant binding interactions with rutin, such as GLU510 in COX-2 and VAL113 and TRP114 in AChE, whereas others, like ILE274 in COX-2 and GLN488 in AChE, showed greater fluctuations due to lesser interactions. Finding from the RMSF values underscored the differential stability conferred by rutin binding, particularly enhancing the stability of AChE, BChE, and COX-2 complexes and its potential to modulate the targets differently.

The rGyr describes the compactness and active site stability of protein-ligand complex 72. Findings from this study revealed that the target-rutin complexes had initial instability across all systems within the first 20 ns, which gradually achieved stability, maintaining a constant state from 60 ns to the end of the simulation. However, the reduced lower mean rGyr of rutin-NOx suggests greater stability and hence the potential of rutin to form compact complex with NOx for modulatory effect. The molecular surface area (MolSA) describes interactions of a molecule with surrounding molecules and environment including its interaction with Vander waal surface and area open to steric hinderance 73. The polar surface area (PSA) describes molecules' exposed surface to polar, charged, or functional groups thus describing the hydrophilic nature of a molecule. The more polar a molecule is, the more difficult it transport across the membrane 74. The observation that the MolSA and PSA plots fluctuated less and had marginally different mean values indicating the formation of stable complexes and ability to interact with surrounding molecules and polar environments. The SASA describes the accessibility of a protein surface area to solvent with lower values indicating greater binding stability 74,75. However, the observed fluctuations in SASA plot for all the complexes indicate the differences in accessibility to solvent. The recorded higher mean SASA value of NOx-rutin complex suggests its reduced accessibility to solvent and stability. The observed deviation in the MolSA, PSA, and SASA values for NOx-rutin complex suggests lower binding interaction of rutin with NOx. This implies that rutin possesses reduced potential for modulating NOx relative to other targets in eliciting anti-inflammatory effects.

A notable limitation of this study is the potential variability in the bioactivity of Hura crepitans extracts, which may arise from ecological factors such as geographic origin, soil composition, and climate conditions that influence phytochemical profiles. These variations underscore the importance of standardizing extraction protocols and characterizing the bioactive compounds to ensure reproducibility. Additionally, while the *in vitro* and in silico methodologies provided valuable insights into the antioxidant and anti-inflammatory properties of the extract and its metabolites, the absence of *in vivo* validation limits the ability to extrapolate these findings to physiological contexts. Future studies should focus on preclinical *in vivo* models to confirm the therapeutic potential of these compounds and further elucidate their pharmacodynamics and safety profiles.

## Conclusions

This study highlights the antioxidant and anti-inflammatory potential of *Hura crepitans* through a combination of *in vitro* and in silico analyses. Key phytochemicals, including epigallocatechin and rutin, were identified as significant contributors to the observed biological activities. The phenolic constituents of CMEHC demonstrated strong binding affinities and stability with key protein targets, suggesting their ability to scavenge free radicals generated during oxidative stress and mitigate inflammatory responses. These findings support the medicinal value of *H. crepitans* and its potential as a source of therapeutic agents. Further *in vivo* and clinical studies are needed to validate its efficacy and safety.

## Figures and Tables

**Table 1:** LC-MS-identified compounds in the crude methanolic extract of *Hura crepitans*

Peak#	Retention time (min)	Area (A) %	Height (H) %	A/H	Base peak (m/z)	Compound	Fragments
1	14.139	5.711837	4.78373	22,002	611.15	Quercetin-3-rutinoside	611, 303
2	21.480	2.42955	3.377835	13,254	338.25	Dihydroberberine	225, 326, 338
3	24.926	0.882143	1.855545	8760	256.30	Hexadecanoic acid	256, 618
4	25.234	18.261	16.49932	20,394	304.25	Epigallocatechin	282, 306
5	26.433	1.151855	1.510291	14,054	284.35	2-(3,4-Diydroxyphenyl)- 5,7-dihydrochromene-4-one	286, 661

Retention time (RT) values correspond to the elution times of the compounds in the LC-MS analysis. Area (A) % and Height (H) % represent the proportional peak area and height, respectively, relative to the total detected peaks, indicating compound abundance. The A/H ratio provides insights into the sharpness of the peak, which is related to compound concentration. Base peak (m/z) refers to the most abundant ion detected for each compound. Fragment ions indicate the specific molecular fragments identified during the analysis, which aid in compound identification.

**Table 2:** Pharmacokinetics, drug-likeness, and physicochemical properties of the constituents of the crude methanolic extract of *H. crepitans*

	Properties	Rutin	Dihydroberberine	Epigallocatechin
Absorption	Papp (Caco-2 permeability)	-6.336 cm/s	-5.446 cm/s	-6.306 cm/s
	Pgp-inhibitor	-(0.002)	+(0.955)	-(0.006)
	Pgp-substrate	-(0.978)	-(0.147)	-(0.003)
	HIA	+(0.925)	+(0.002)	+(0.274)
	Bioavailability	0.17	0.55	0.55
Distribution	PPB	83.81%	96.53%	91.16%
	VD	0.754 L/kg	0.839 L/kg	0.572 L/kg
	BBB	-(0.111)	+(0.558)	-(0.6750)
Metabolism	CYP1A2 inhibitor	-(0.013)	+(0.954)	-(0.905)
	CYP1A2 substrate	0.026	0.92	0.124
	CYP2C9 inhibitor	-(0.002)	-(0.614)	-(0.174)
	CYP2C9 substrate	-(0.246)	-(0.914)	-(0.52)
	CYP2C19 inhibitor	-(0.011)	+(0.967)	-(0.002)
	CYP2C19 substrate	(0.05)	0.793	0.051)
	CYP2D6 inhibitor	-(0.007)	+(0.919)	-(0.037)
	CYP2D6 substrate	-(0.155)	+(0.931)	+(0.224)
	CYP3A4 inhibitor	-(0.013)	+(0.956)	-(0.142)
	CYP3A4 substrate	+(0.003)	+(0.682)	-(0.150)
Elimination	Half-life time (T ½)	0.524 h	0.395 h	0.87 h
	Clearance rate	1.349	12.989 mL/min/kg	17.081 mL/min/kg
Toxicity	AMES	+(0.805)	+(0.818)	-(0.437)
	LD_50_	5000 mg/kg	350 mg/kg`	10000 mg/kg
	Drug likeness (Lipinski)	No	Yes	Yes
	Lead likeness	1	1	0
	Pains	1 alert	0 alert	1 alert
Physicochemical properties	Molecular weight	610.52	337.37	306.27
	HB acceptor	16	4	7
	HB donor	10	0	6
	TPSA	269.43	40.16	130.61
	Log P	-0.763	4.158	0.736
	Log S	-3.928	-5.267	-2.88
	LogD	0.695	3.602	0.905
	Synthetic accessibility	6.52	3.42	3.53

HIA, human intestinal absorption; PPB, plasma protein binding; PgP, P-glycoprotein; LD50, acute toxicity; CYP, cytochrome P450; Caco-2, colorectal adenocarcinoma cells; BBB, blood-brain barrier; HB, hydrogen bond+, positive result; -, negative result; VD, volume distribution; CL, clearance rate; LogD, distribution coefficient D; LogP, distribution coefficient P

**Table 3:** Binding energies (ΔG) of quercetin-3-rutin (rutin), epigallocatechin, and dihydroberberine calculated as free binding energies (kcal/mol)

Target protein	ΔG (kcal/mol)^ a^				
	Rutin	Epigallocatechin	Dihydroberberine	Ascorbic acid	Aspirin
COX-2	-10.9	-8.4	-7.9	-	-7.2
AChE	-9.9	-8.7	-6.0	-	-7.0
BChE	-10.9	-7.2	-7.3	-	-6.6
NF-κB	-7.2	-5.9	-6.5	-	-6.9
Nox	-9.0	-8.0	-7.7	-	-6.6

^a^ The lowest free binding energies were calculated by the AutoDock Vina program. COX, cyclooxygenase; AChE, acetylcholine esterase; BChE, butyrylcholinesterase; NF, nuclear factor; Nox, NADPH oxidase

**Table 4:** Protein-ligand interaction analysis of rutin, epigallocatechin, and dihydroberberine

Target protein	Rutin	Epigallocatechin	Dihydroberberine
	Interacting amino acid residues	Interacting amino acid residues	Interacting amino acid residues
COX-2	^a^(HIS39A, PRO40A, CYS41A, MET48A, TYR136A, VAL155A, ASP157A, GLU322B, TRP323B, GLY324B), ^b^(PRO154A, ASP158A, GLN327B), ^b,c^(PRO153A), ^b,f^(ASN34A), ^b,h^(SER49A), ^g^(GLY135A), ^c^(CYS36A, PRO156A), ^f^(GLN461A), ^j^(CYS47A)	^a^(HIS39A, PRO40A, CYS41A, GLY45A, GLY135A, LYS137A, PRO154A, GLN461A), ^b^(TYR130A),^ c^(VAL46A, PRO153A), ^d^(CYS36A), ^c,d,h^(CYS47A), ^e^(PRO156A), ^f^(ASN34A)	^a^(ASP133B, GLY135B, TYR136B, GLU326A, GLN327A), ^d^(CYS36B, PRO154B), ^c,d^(PRO156B), ^g^(ASP157B), ^g,h^(ASN34B)
AChE	^a^(ASP72A, TRP84A, ASN280A, LEU282A, PHE284A, ASP285A, ILE287A, PHE290A, PHE331A), ^b^( PHE288A, ARG289A),^ b,f^(SER286A), ^b,l^(TRP279A), ^h^(TYR121A), ^h,k^(TYR70A), ^k^(PHE330A, TYR334A)	^a^(TYR70A, ASP72A, TYR121A, TRP279A, LEU282A, SER286A, ILE287A, PHE288A, PHE290A, PHE331A, HIS440A), ^b^(ARG289A),^ c^(TYR334A), ^h,k^(PHE330A)	^a^(LYS410A, THR497A), ^b^(ASN525), ^c^(HIS406A), ^d^(VAL518A, CYS521A, VAL522A), ^h^(ASN409A)
BChE	^a^(ASN68A, ILE69A, SER79A, GLY115A, GLY116A, GLY117A, THR120A, GLY121A, LEU125A, SER198A, PRO285A, LEU286A, VAL288A, TRP430A, MET437A, GLY439A, ILE442A), ^b^(GLY78A, ASN83A, TYR128A, GLU197A, TYR440A),^ b,g^(HIS438A), ^b,l^(TRP82A), ^c^(ALA328A, TYR332A), ^f^(ASP70A), ^l^(PHE329A)	^a^(ASP70A, GLY78A, TRP82A, GLY117A, GLU197A, LEU286A, SER287A, PHE329A, TRP430A, MET437A, HIS438A, GLY439A, TYR440A), ^b^(PRO285A), ^c,d^(ALA328A),^ c,e,l^(TYR332A)	^a^(PRO230A, TRP231A, ARG242A, PRO281A, GLY283A, SER287A, VAL288A, PHE357A, PHE358A, GLY360A), ^b,e^(THR284A), ^c^(PRO359A, TYR396A), ^d^(VAL280A), ^g^(LEU286A, ASN397A), ^l^(TYR282A)
NF-κB	^a^(GLY52B, PHE53B, HIS64B, GLY66B, PRO68B, GLU73B, LYS74B, LYS77B, TYR79B, SER240B, ASN247B), ^b^(GLY65B, SER72B),^ f^(LYS49B), ^m^(SER63B)	^a^(ARG56A, VAL58A, GLU60A, GLY113A, LYS114A, GLY138A), ^b^(TYR57A, GLY61A, GLY65A, ASN136A), ^c,d^(PRO62A, VAL112A),^ c^(LEU140A), ^g^(SER63A)	^a^(GLY133A), ^c^(LYS114A, PHE134A), ^d^(CYS116A, CYS121A, ALA135A),^ c,d^(VAL123A), ^c,l^(HIS115A), ^g^(ASN136A)
NOx	^a^(ILE4A, TYR201B, GLU203B, HIS338B, HIS354B, ILE355B, VAL358B, GLY359B, TRP361B, THR362B, ILE411B, GLY412B, PRO415B, PHE451B), ^b^(THR341B, ARG356B, THR414B),^ c,g^(PRO339B, PHE340B), ^f^(ASP360B), ^k^(PHE202B)	^a^(ILE67B, ALA105B, TRP106B, ILE189B, ILE190B, SER193B, HIS209B), ^b,c^(LYS102B), ^c^(HIS101B),^ c,d^(LEU98B), ^e^(VAL71B), ^c,l^(PHE205B), ^f^(ARG198B)	^a^(SER77B, GLY81B, ASP95B, PHE391B), ^b,c,d,g^(ARG80B), ^c,d^(PRO390B)

^a^ van der Waals, ^b^ conventional hydrogen bonds, ^c^ Pi-alkyl, ^d^ alkyl, ^e^ Pi-sigma, ^f^ unfavorable donor-donor, ^g^ carbon hydrogen bonds, ^h^ Pi-donor hydrogen bonds, ^i^ Pi-anion, ^j^ Pi-sulfur, ^k^ Pi-Pi stacked, ^l^ Pi-Pi T-shaped, ^m^ unfavorable acceptor-acceptor. COX, cyclooxygenase; AChE, acetylcholine esterase; BChE, butyrylcholinesterase; NF-κB, nuclear factor-κB; Nox, NADPH oxidase

**Table 5:** Binding energies of quercetin-3-rutin (rutin), epigallocatechin, and dihydroberberine calculated as free binding energies (kcal/mol)

	Rutin		Epigallocatechin		Dihydroberberine	
	Docking score	MMGBSA	Docking score	MMGBSA	Docking score	MMGBSA
NF-kB	-10.6959	-61.6574	-7.12783	-34.6015	-2.49564	-35.8014
COX-2	-13.9397	-43.3774	-7.2927	-34.2378	-6.8767	-36.5534
Nox	-6.86932	-38.1605	-4.81111	-29.0129	-4.08969	-47.3654
AChE	-12.4512	-51.0907	-11.1507	-20.3044	-8.13601	-37.8667
BChE	-15.0882	-41.2449	-9.3766	-33.8217	-6.17575	-31.2929

**Table 6:** Molecular dynamic properties of rutin and target protein complexes after a 100-ns simulation

Target	RMSD (Å)	RMSF (Å)	rGyr (Å)	MolSA (Å^2^)	PSA (Å^2^)	SASA (Å^2^)
COX-2	2.06±0.25	1.01±0.39	4.96±0.09	457.99±7.99	446.99±16.70	251.77±27.02
AChE	1.31±0.12	0.71±0.38	4.67±0.12	463.94±8.85	450.55±13.54	147.02±27.10
BChE	1.55±0.24	0.77±0.48	4.42±0.05	440.65±6.041	449.17±8.64	75.09±20.65
NOx	4.68±0.76	1.89±0.87	4.41±0.22	438.26±18.16	433.79±17.39	496.11±109.47
NF-kB	5.67±0.70	1.74±0.72	4.45±0.19	452.69±19.30	469.08±16.31	385.17±53.16

Values are presented as the mean±standard deviation (100 ns). RMSD, root mean squared distance; RMSF, root mean squared fluctuation; rGyr, radius of gyration; MolSA, molecular surface area; PSA, polar surface area; SASA, solvent-accessible surface area.
